# Protein extraction from Buckwheat, *Chondrus crispus*, and Spelt and assessment of nutritional benefits and limitations in vitro

**DOI:** 10.1038/s41538-025-00540-6

**Published:** 2025-09-24

**Authors:** Ethan Cain, Suzanne M. Hodgkinson, Warren McNabb, André Brodkorb, Linda Giblin, Maria Hayes

**Affiliations:** 1 Riddet Institute, Massey University, Palmerston North, New Zealand; 2 Department of Food BioSciences, Teagasc Food Research Centre, Ashtown, Dublin D15 DY05 Ireland; 3 Teagasc Food Research Centre, Moorepark, Fermoy, Co Cork P61 C996 Ireland

**Keywords:** Biotechnology, Agriculture

## Abstract

Plant protein consumption has increased globally but concerns exist regarding their ability to provide sufficient amino acids to consumers. Extraction methods that can separate protein from anti-nutritional factors have potential to increase the nutritional value of this biomass. Few studies concerning analysis of the amino acid content of plant protein extracts exist. In this work, three different protein extraction methods were used to generate protein extracts from Buckwheat, Spelt and the red seaweed *Chondrus crispus*. Methods used include ultra-sonication in water combined with ammonium sulphate-induced protein precipitation; an enzymatic extraction method using the enzymes Alcalase and Viscozyme, and an iso-electric precipitation extraction method using alkaline protein solubilization followed by acidic protein precipitation. Proteins extracted using the enzymatic method contained the highest proportion of essential amino acids (EAA) in viable quantities, and the method holds promise for use in the generation of alternative marine and cereal protein extracts for human consumption.

## Introduction

As the global population increases and climate conditions continue to threaten global food security^1^ there is a greater need to find novel ways of providing adequate nutrition for the human population. This is particularly important as consumers continue to select and increase the proportion of novel, speciality foods in their diets in response to animal welfare and climate change concerns^2^. In parallel, demand for dietary protein continues to increase and this has led to a focus on alternative resource use for protein extraction^3^.

Protein extraction can increase protein concentration^4^, protein digestibility^5^ and protein solubility^6^, and decrease the amounts and activities of anti-nutritional factors^7^. Increased protein digestibility of proteins comes at the expense of other essential nutrients such as carbohydrates. This study examines the use of three different protein extraction methods and production of protein extracts at lab-scale from biomass including Buckwheat *(Fagopyrum esculentum)*, the seaweed Irish moss (*Chondrus crispus* *(Linnaeus) J.Stackhouse*), and Spelt *(Triticum spelta)*.

Buckwheat is a pseudo-cereal with a protein content ranging from 8% to 18% on a dry matter basis, depending on cultivar^8^. The protein extracts derived from Buckwheat had complete distribution of amino acids containing all the required dietary essential amino acids^9^. Buckwheat proteins can broadly be subtyped as albumins, globulins, glutelins and prolamins^10^. *Chondrus crispus* is a red seaweed with a reported protein content ranging from 19 to 35% of the dry matter content of biomass^11^ and containing all the required essential amino acids^12^. Additionally, *C. crispus* is harvested for use as a gelling agent, due to its content of carrageenans. As an ancient grain, Spelt contains between 14 and 20% protein, based on dry matter content^13^. Spelt is more sustainable with lower soil nitrogen requirements compared to common grains such as wheat, and spelt proteins can broadly be subtyped as glutenins, gliadins, albumins and globulins^14^.

The major challenge concerning protein extraction from plant and algal material is the requirement to break down structures within the cell wall for protein release. Plant and algae cell walls are composed of cellulose, hemicellulose, pectins, and in the case of seaweeds carrageenan^4^. Additionally, plant material contains anti-nutritional factors such as fibre, protease inhibitors, saponins and phenolics that pose challenges concerning the nutritional quality of proteins^15^. Anti-nutritional components limit the digestibility of proteins and either physically preventing interactions between proteins and digestive enzymes, or chemically inhibiting the enzymatic digestion of proteins by conjugating with protein^16^. Therefore, the primary focus of this study was to evaluate the effectiveness of three protein extraction methods applied to selected biomasses of terrestrial and marine origin. These methods were the “Salt & Sonic” method, an enzymatic protein extraction method and an Iso-electric precipitation method.

Proteins extracted from Spelt, Buckwheat and *C. crispus* were analysed in two ways. The effectiveness of the extraction method applied independently to the biomass was determined by how well the extraction method could separate protein from the non-protein components and concentrate protein in the extract. To eliminate the potential of interference from non-protein compounds and overestimation of protein content in the final extract, the protein content of extracts was evaluated using three different methods of protein quantification.

In addition, the protein quality of extracts generated using the different extraction processes was determined. Protein quality is an evaluation of how well a protein provides the necessary essential amino acids (EAA) to fulfil dietary requirements. The content of EAA within the protein extract was examined and compared to the EAA content of the starting biomass. A general increase in EAA content increases the degree to which the protein is able to satisfy nutritional requirements. Additionally, the EAA content of protein extracts was compared to a nutritional requirement-scoring pattern to determine the potential of the extract to satisfy nutritional requirements.

## Results

### Extraction Yields

Enzymatic hydrolysis resulted in protein yields from Buckwheat and *C. crispus* of 24.7 ± 0.4% and 50.8 ± 3.6% protein, respectively. Yields were greater than those resulting from the salt & sonic and iso-electric extraction methods applied to the same biomass (Table 1). The extraction yield of protein from Spelt using the salt and sonic extraction method and the enzymatic extraction method were not significantly different (*P* ≥ 0.05). However, a greater yield of protein was obtained with both the enzymatic and iso-electric extraction methods compared to protein yield obtained with the iso-electric precipitation method applied to Buckwheat and *C. crispus* (20.8 ± 0.5% available protein and 4.91 ± 0.1%). When the enzymatic extraction method was applied to Buckwheat, a volume of 4.7 g of protein extract was obtained from 20 g of the initial starting biomass. In the case of *C. crispus*, from 20 g of biomass, the greatest protein extract volume was obtained using enzyme extraction, where a protein extract yield of 8.9 g was achieved. In the case of Spelt, from an initial starting biomass quantity of 20 g, 4.2 g of protein extract resulted from when the Salt & Sonic extraction method was applied to biomass.

**Table 1:** Protein Mean Dry Matter Protein Extraction Yields obtained using the Sonic & Salt, Enzymatic, and Iso-Electric Protein extraction methods (independently, *n* = 3) applied to biomass & determined via amino acid nitrogen analysis (nitrogen conversion factor 6.25)

Source Biomass	Sonic & Salt Extraction	Enzymatic Extraction	Iso-Electric Extraction
**Buckwheat**	6.40 ± 0.01 (%)^**BC**^	24.77 ± 0.40 (%)^**AC**^	16.98 ± 0.01 (%)^**AB**^
***C.crispus***	4.48 ± 0.00 (%)^**B**^	50.84 ± 3.62 (%)^**AC**^	4.59 ± 0.00 (%)^**B**^
**Spelt**	19.98 ± 1.41 (%)^**C**^	20.82 ± 0.48 (%)^**C**^	4.91 ± 0.00 (%)^**B**^

^**A**^: P < 0.05 vs Sonic & Salt Extraction^**; B**^: P < 0.05 vs Enzymatic Extraction^**; C**^: P < 0.05 vs Iso-Electric Extraction.

### Protein Extract Composition Relative to Crude Biomass

**Fig. 1:**
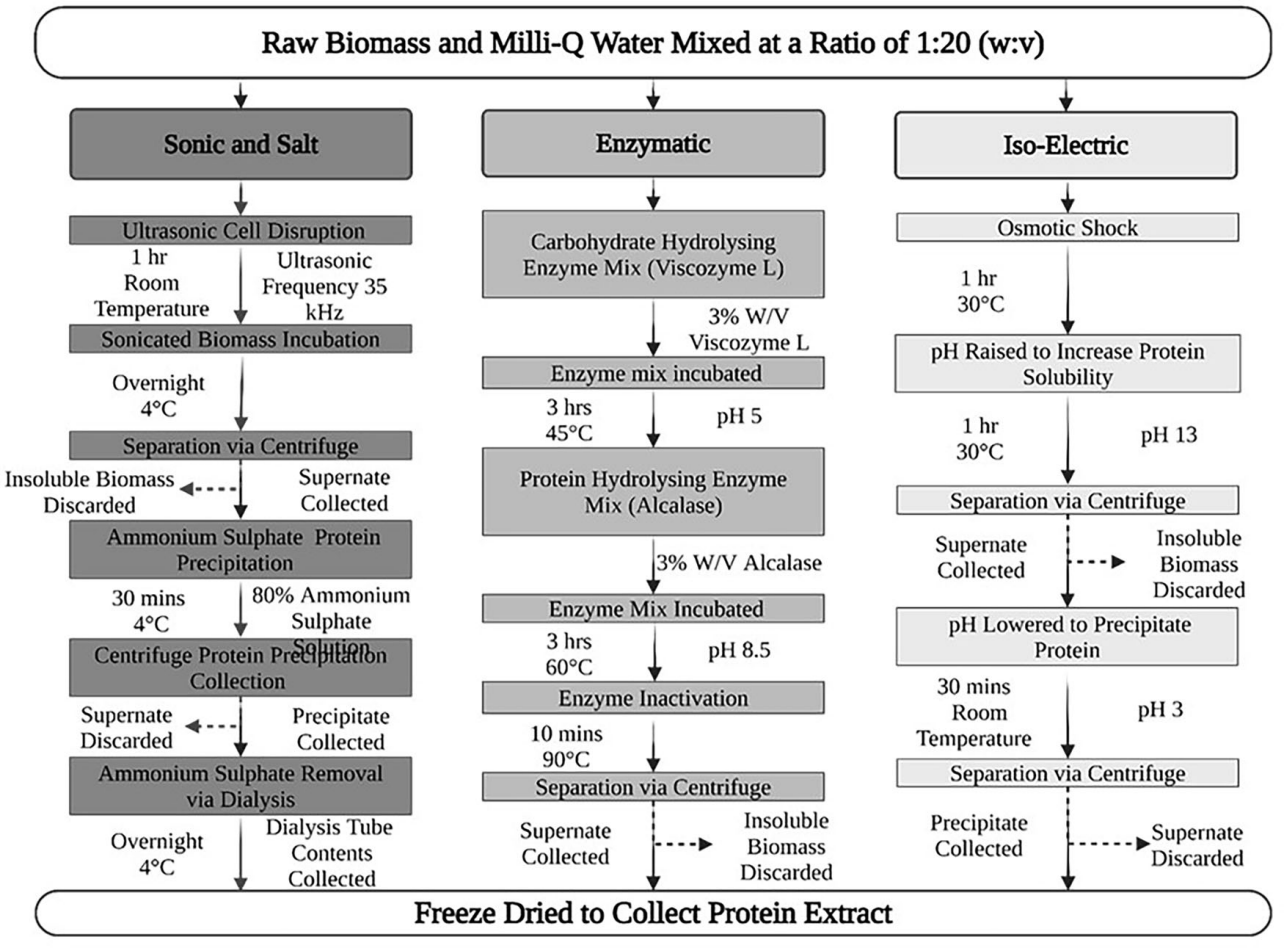
Schematic representation of the three different extraction processes applied to biomass to generate protein extracts.

Proteins were extracted as outlined in Fig. 1.

**Fig. 2:**
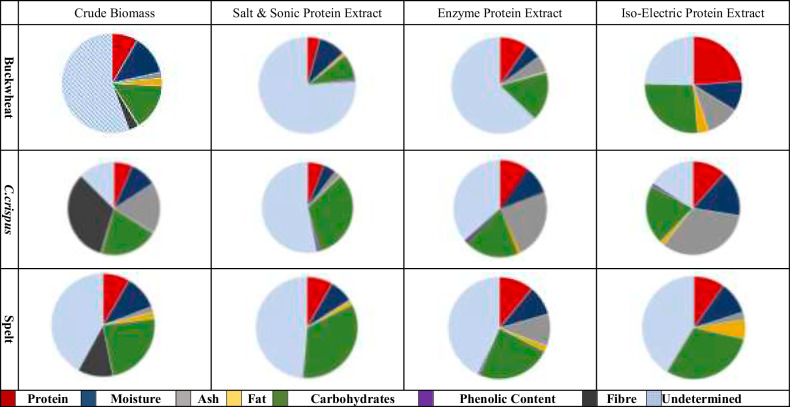
Proximate compositional analysis of source biomass and generated protein extracts. To determine the composition of the source biomass and the resulting protein extracts, the contents of each major nutritional factor was determined. each pie wedge represents the percentage (%) composition of the component from total. compositional analysis was performed in triplicate. The protein fraction represents protein content as determined using the Amino Acid Nitrogen (AAN) method. Moisture, Ash, Fat, carbohydrates, and Phenolic content was determined using the method described in materials and methods section. Undetermined fraction is assumed to be carbohydrates based on the standard method of carbohydrate determination as outlined by the United States food and drug administration.

The results from the nutritional analysis of the extracts are shown (Fig. 2).

Overall, very limited selectivity in the extraction resulted with the three extraction processes. An example of this is the Spelt extract, in which the protein content as determined via amino acid analysis as outlined in section “**Protein quality determination”** of the crude biomass (8.3% protein) is the same as the extract generated using the salt & sonic method (8.3% protein). The protein content of the enzymatic extract (11% protein) and iso-electric extracts (9.7% protein) are not statistically significantly different (*P* ≥ 0.05) from the protein content of the starting biomass based on the analysis performed in sections 3.1 and 3.3 (compositional analysis section). However, protein extracted using the iso-electric method, applied to Buckwheat contained twice as much protein (23.7% protein) compared to whole Buckwheat (8.3% protein). In the case of *C. crispus*, the protein content of extracts generated using the salt & sonic extraction were lowest (5.8% protein) and highest for proteins extracted using iso-electric precipitation (11.6% protein). Enzymatic extraction applied to *C. crispus* increased the protein content of extracts from 8.6% protein (raw seaweed) to 10.1% protein in the extract. However, the enzymatic hydrolysis extraction method also resulted in an increase in ash content in the protein extracts (Buckwheat 1.9% to 5.4% for protein extract, *C. crispus* 23.8% to 23.4% for protein extract and 1.7% to 10.3% for the Spelt protein extract). In the case of iso-electric, precipitation there was also an increase in ash content. The ash content of Buckwheat increased from 1.9% for whole Buckwheat to 11.1% for the protein extract, *C. crispus* increased from 23.7% for whole biomass to 33.1% ash in the protein extract and Spelt ash content increased from 1.7% for whole Spelt to 2.4% for the spelt protein extract, respectively. In both the salt & sonic and enzymatic extractions, the moisture content of freeze-dried powders decreased compared to the moisture content of the raw biomass. However, in the case of *C. crispus*, iso-electric precipitation resulted in an increase in moisture content of protein extracts compared to the crude biomass (12.5% & 16.0%, respectively).

### Protein concentration determination

The protein content of the sonic & salt, enzymatic, and iso-electric protein extracts generated from Buckwheat, *C. crispus*, and Spelt determined using the amino acid nitrogen (AAN) and Dumas methods are shown in Table 2. For buckwheat, the protein content of extracts generated using the sonic & salt method was determined as 453.75 mg/g of protein using the Dumas method. The protein content of extracts generated from *C. crispus* was greatest when the sonic & salt extraction method was applied with extracts containing 567.08 mg/g protein as determined using the Dumas method. For Spelt, the salt & sonic extraction method resulted in extracts containing 461.80 mg/g protein determined using the Dumas method. There is a significant difference (*P* < 0.05) in protein content, depending on the protein analysis method used. For example, there is a ten-fold difference reported in the protein content of the Buckwheat salt & sonic protein extract depending on whether the AAN method of analysis was used for protein determination (453.8 ± 111.9 mg/g v’s 46.5 ± 0.1, respectively). These differences are highlighted in Table 2.

**Table 2:** Results for protein content determination Comparing direct and indirect measurement of protein content. Mean dry matter protein content is expressed in mg/g

Source Biomass	Method of protein determination	Crude Biomass Protein (mg/g)	Sonic & Salt Extraction Protein (mg/g)	Enzymatic Extraction Protein (mg/g)	Iso-Electric Extraction Protien (mg/g)
**Buckwheat**	**Dumas**	148.95 ± 12.26 ^**A**^	453.75 ± 117.86^**B**^	127.54 ± 7.36 ^**A**^	257.68 ± 69.69 ^**C**^
	**Amino Acid Nitrogen**	110.44 ± 18.18 ^**A**^	51.55 ± 7.13 ^**A**^	100.41 ± 1.59 ^**A**^	251.05 ± 18.17^**B**^
***C.crispus***	**Dumas**	80.73 ± 0.25 ^**A**^	567.08 ± 10.66^**B**^	88.81 ± 6.96 ^**A**^	95.38 ± 6.09 ^**A**^
	**Amino Acid Nitrogen**	116.43 ± 15.93 ^**A**^	65.47 ± 7.07^**B**^	111.91 ± 7.99 ^**A**^	103.36 ± 26.51 ^**A**^
**Spelt**	**Dumas**	109.57 ± 3.12 ^**A**^	461.80 ± 185.68^**B**^	163.27 ± 17.53 ^**A**^	363.60 ± 26.83^**B**^
	**Amino Acid Nitrogen**	110.64 ± 7.64 ^**A**^	89.51 ± 6.31 ^**A**^	121.94 ± 2.77 ^**A**^	113.00 ± 6.14 ^**A**^

Protein to nitrogen conversion factor for Dumas method: Spelt, 5.54^36^, *C. crispus*, 3.55^12^, Buckwheat, 5.94^36^. The Protein to nitrogen conversion factor for amino acid nitrogen: 6.25.

Matching letters indicate no statistically significant difference when compared using the same source biomass.

^A^(***P*** < 0.05) compared to crude biomass protein.

^B^(***P*** < 0.05) compared to sonic & salt extraction protein.

^C^(***P*** < 0.05) compared to iso-electric extraction protein.

### Protein quality determination

The enzymatic extraction method produced extracts with the highest overall content of EAA (Buckwheat: 38.01 mg/g, *C. crispus:* 37.34 mg/g, Spelt 38.10 mg/g). There was a significant (P < 0.05) increase in the EAA: TAA ratio of protein extracts compared to the whole biomass. For example, the EAA percentage of protein extracts of Buckwheat increased from 39.1% for the whole biomass to 44.0% for the protein extract. For Spelt the percentage EAA content increased from 32.0% for whole Spelt to 37.5% EAA content in the protein extract, respectively. For Buckwheat, there were significant decreases (*P* < 0.05) in amino acid content of protein extracts generated using the Salt & sonic method and the EAA content reduced from 30.79 mg/g EAA for whole biomass to 14.16 mg/g EAA for protein extract and the TAA content reduced from 78.97 mg/g for whole biomass to 36.60 mg/g EAA content in the Buckwheat protein extract. The EAA percentage and EAA: TAA ratio increased when isoelectric precipitation was applied to Buckwheat (the EAA increased from 30.79 mg/g in whole buckwheat to 77.75 mg/g in the buckwheat protein extract while the TAA content increased from 78.97 mg/g in Buckwheat to 200.90 mg/g in the buckwheat protein extract). This, however, did not result in a significant change in the percentage content of EAA. In the case of *C. crispus*, the salt & sonic extraction protocol also significantly decreased (*P* < 0.05) amino acid content (EAA, *C. crispus* whole - 33.71 mg/g to 22.97 mg/g in *C. crispus* protein extract, TAA 82.19 mg/g in *C. crispus* whole to 54.40 mg/g in *C. crispus* protein extract). In the case of the iso-electric precipitation extraction protocol, there was a significant (P < 0.05) decrease in the percentage of EAA from 41.1% in *C. crispus* whole to 38.2% in the *C. crispus* extract with non-statistically significant differences in the content of individual amino acids. This change in amino acid content also resulted in a significant change (*P* < 0.05) in amino acid content of Spelt extract where the percentage of EAA increased from 32.0% to 38.1% following iso-electric precipitation of spelt. Changes to individual amino acids content in each extract and source biomass are described in Supplemental Table 1, supplemental Table 2, supplemental Table 3.

### Nutritional requirements

**Fig. 3:**
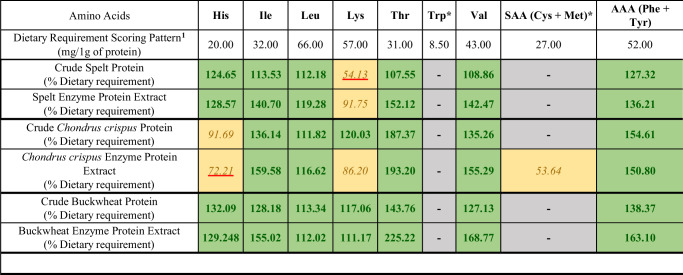
^1^Scorring pattern: Child (6 months to 3 years) FOOD AND AGRICULTURE ORGANIZATION OF THE UNITED NATIONS,ROME, 2013. Bold Values indicate greater than 100% Dietary Requirements. *Italicised* values indicate results less than 100% Dietary Requirements. - Values indicate that no value was reported or that not all replicates reported a value. Underline indicates potential to be the limiting amino acid. *, Indicate amino acids that are likely to be degraded because of hydrolysis conditions.

From Table 3, the enzymatic protein extracts have the highest EAA content expressed as milligrams per gram and, in general, the greatest EAA content in TAA (Fig. 3).

**Table 3:** Amino Acid Content of biomass and extracts generated from the same

Source Biomass	Extraction Method	∑EAA (mg/g DM)	∑TAA (mg/g DM)	EAA (%)
**Buckwheat**	Crude Biomass Protein	30.79 ± 4.04	78.97 ± 12.4	39.1% ±1.00
	Salt & Sonic Protein	14.16 ± 0.01*	36.60 ± 0.09*	38.7% ±0.07
	Enzyme Protein	38.01 ± 0.74*	86.39 ± 1.46	44.0% ±0.10*
	Iso-Electric Protein	77.75 ± 0.00*	200.90 ± 0.00*	38.7% ±0.00
***C.crispus***	Crude Biomass Protein	33.71 ± 0.94	82.19 ± 6.65	41.1% ± 1.10
	Salt & Sonic Protein	22.97 ± 0.00*	54.40 ± 0.00*	42.2% ±0.00
	Enzyme Protein	37.34 ± 2.81	90.81 ± 6.67	41.1% ±0.20
	Iso-Electric Protein	35.71 ± 0.00	93.54 ± 0.00	38.2% ±0.00*
**Spelt**	Crude Biomass Protein	28.90 ± 0.94	90.03 ± 0.29	32.0% ±1.00
	Salt & Sonic Protein	27.62 ± 1.76	83.87 ± 5.87	33.9% ±0.21
	Enzyme Protein	38.10 ± 0.81*	101.67 ± 2.6	37.5% ±0.90*
	Iso-Electric Protein	32.98 ± 0.00	86.55 ± 0.00	38.1% ±0.00*

* *P* < 0.05 when compared to Crude Biomass as determined via two-way ANOVA.

The Buckwheat protein generated using the enzymatic protocol contains greater than 100% of dietary requirements of each essential amino acid. Conversely, *C. crispus* has the highest number of limiting amino acids (histidine 72.21% and lysine 86.20%) with two of the detected amino acids providing less than 100% of dietary required amino acids. Nutritional analysis for all other extracts can be found in supplemental Table 4.

### Anti Nutritional Factors

From Table 4, there were significant differences (*P* < 0.05) in the total phenolic content of the crude biomass and the protein extracts. The sonic & salt extraction method resulted in a significant increase (*P* < 0.05) in total phenolic content in protein extracts compared to the crude biomass for all selected biomass types (Buckwheat: 3.39 mg(GAE)/g vs 7.12 mg(GAE)/g, *C. crispus*: 5.44 mg(GAE)/g vs 10.10 mg(GAE)/g, Spelt: 0.15 mg(GAE)/g vs 4.42 mg(GAE)/g). *C. crispus* protein extracts generated with both enzymatic and iso-electric precipitation protocols contained significantly (*P* < 0.05) greater total phenols compared to the crude biomass but not significantly different when compared to each other (5.44 mg(GAE)/g vs 9.57 mg(GAE)/g and 10.73 mg(GAE)/g, respectively). Protein extracts generated from Buckwheat and Spelt generated using enzymatic hydrolysis and iso-electric precipitation had phenolic contents significantly different to each other (Buckwheat: 3.22 mg(GAE)/g vs 1.61 mg(GAE)/g and Spelt: 6.58 mg(GAE)/g vs 1.78 mg(GAE)/g) but not when compared to the crude biomass.

**Table 4:** Total Phenolic Content of Protein Extracts and Crude Biomass

Source Biomass	Crude Biomass (mg(GAE)/g)	Sonic & Salt Extraction (mg(GAE)/g)	Enzymatic Extraction (mg(GAE)/g)	Iso-Electric Extraction (mg(GAE)/g)
**Buckwheat**	3.39 ± 0.14^**B**^	7.12 ± 2.17^**ACD**^	3.22 ± 0.50^**BD**^	1.61 ± 0.38^**BC**^
***C.crispus***	5.442 ± 2.00^**BCD**^	10.10 ± 1.20 ^**A**^	9.57 ± 0.78 ^**A**^	10.37 ± 0.93 ^**A**^
**Spelt**	0.15 ± 0.00^**BC**^	4.42 ± 0.56^**ACD**^	6.58 ± 0.44^**ABD**^	1.78 ± 0.37^**BC**^

The average total phenolic content represented as milligrams of Gallic acid equivalents (GAE) per grams of extract ± SD.

^A^*P* < 0.05 vs crude biomass.

^B^*P* < 0.05 vs sonic & salt extraction.

^C^*P* < 0.05 vs enzymatic extraction.

^D^*P* < 0.05 vs Iso-electric extraction.

### Carbohydrate Content

Extracts generated using enzymes contained the least carbohydrates relative to the crude biomass. As shown in Table 5, the salt and sonic extractions increased the carbohydrate content of extracts relative to the whole biomass in the case of *Spelt* and *C. crispus*. In the case of Buckwheat, there is a statistically significant (*P* < 0.05) difference between the carbohydrate content of the salt & sonic protein extract and the iso-electric protein extract (8.72% vs 26.52%).

**Table 5:** The carbohydrate content of protein extracts and crude biomass

Source Biomass	Crude Biomass (%)	Sonic & Salt Extraction (%)	Enzymatic Extraction (%)	Iso-Electric Extraction (%)
**Buckwheat**	15.08 ± 3.01	8.73 ± 1.77^**B**^	16.50 ± 9.91	26.25 ± 1.85^**A**^
***C.crispus***	27.19 ± 3.166	32.87 ± 4.10	18.94 ± 9.43	20.77 ± 5.08
**Spelt**	24.00 ± 11.15	33.60 ± 4.51	23.87 ± 8.71	30.13 ± 7.04

Mean carbohydrate content represented as percentage of crude biomass± SD.

^A^*P* < 0.05 vs sonic & salt extraction.

^B^*P*  < 0.05 vs Iso-electric extraction.

### Trypsin activity inhibition

**Fig. 4:**
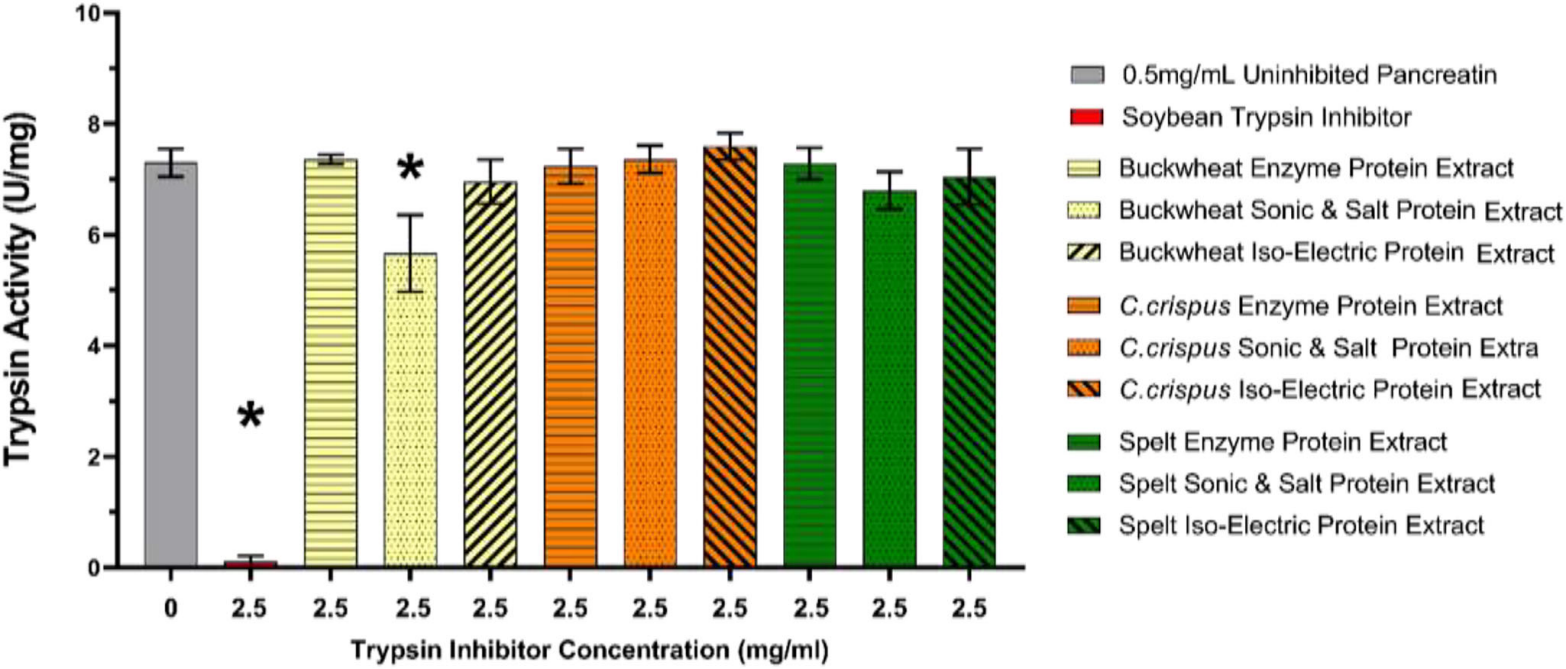
Inhibition of trypsin by protein extracts generated from Buckwheat, *C. crispus* and Spelt.

Trypsin activity is depicted in Fig. 4.

Protein extracted from Buckwheat using the salt & sonic protocol had a significant (*P* < 0.05) impact on trypsin activity. This extract caused a 25% reduction in trypsin activity compared to uninhibited pancreatin. In the case of all other extracts, there was no significant difference in trypsin activity observed.

## Discussion

The primary focus of this study was to evaluate the effectiveness of three different methods of protein extraction on three different biomasses. The results demonstrate how an extraction method may improve the nutritional quality of proteins from Buckwheat, *C. crispus* and Spelt.

Protein extraction yield is an important factor as it is the main determinant of the efficiency of the extraction process. Results demonstrate that the best yield of protein was obtained from the biomasses using enzymatic hydrolysis (Buckwheat: 27% protein yield, *C. crispus*: 52% protein yield and Spelt 21% protein yield, respectively). The major limiting factor on the availability of protein from the three-biomass types is the stiff, carbohydrate structure present in the plant cell wall^17^. The cell wall prevents entry of proteins into solution during the extraction process. Use of the carbohydrate hydrolysing enzyme mixture Viscozyme L during enzymatic extraction helps break down the carbohydrate structure of the cell wall^18^. The salt & sonic method and the iso-electric precipitation method also affect the cell wall structures of the biomass and enhance protein availability. The use of enzymes for extraction also increases the solubility of resulting proteins as the proteolytic enzyme Alcalase breaks down proteins into peptides and free amino acids, which have greater solubility than large proteins^19^. The salt & sonic extraction method may also result in more soluble proteins but the randomness that results from ultrasonic cavitation limits the reliability with which specific protein fragments can be extracted or that all extraction runs will be equally soluble^20^. Isoelectric precipitation is also less likely to improve overall protein extract solubility as the effects of pH on proteins leads to changes in protein folding and confirmations. These may be more soluble, but may also form aggregates, especially following freeze-drying of the extracts^6^.

Protein concentration is also a major component for determining the uses for a protein extract. In the case of extracts present in this study all extracts exhibited very little protein concentration. This will limit the potential uses of any protein extract as the non-protein content can produce undesirable qualities. A prime example of this is the gelling property of the *C.crispus* extracts, the ability to form a highly water absorbent gel limits the ability to produce large quantities of protein extract and can limit the uses cases the extract in liquid beverages as the degree to which the extract needs to be diluted to overcome the gel formation severely limits the maximum amount of protein that the extract could supplement to a protein enriched beverage. A higher concentration of protein was expected in the extracts compared to the starting biomass than observed in this study.

All extracts generated using the enzymatic or the salt & sonic methods had increased protein content relative to the whole, crude biomass as determined by amino acid nitrogen determination of protein content. However, isoelectric precipitation of the proteins resulted in the greatest difference in protein composition relative to the starting biomass. Additionally, a major difference between the composition of the whole biomass and the extracts was the observed increased ash content in extracts generated with the enzymatic and isoelectric precipitation methods. This increase is likely due to the addition of NaOH and HCl to control optimum conditions for enzymatic hydrolysis. Both the enzymatic extraction and iso-electric precipitation methods increase the total salt (NaCl) content because of Na and Cl added for pH adjustments. The iso-electric precipitation method also is likely to reduce protein quality through degradation of proteins. Protein degradation occurs due to exposure to high pH for extended periods of time causing a breakdown of protein both in terms of macro structure folding and sub-unit binding^21^.

Methods for protein quantification have limitations that may affect the accuracy of reported protein content^22^. The differences in indirect and direct measurement of protein content can lead to major differences in reported protein contents of extracts. This is shown most dramatically in the salt & sonic method as it uses ammonium sulphate as a part of the extraction process, which likely increases the total nitrogen concentration within the sample. This may, in part, explain the higher protein content values observed for extracts when the Dumas protocol was employed (Buckwheat: salt & sonic: 45.3%, *C. crispus*: salt & sonic: 56.7%, Spelt: salt & sonic: 46.2%). The ammonium sulphate content likely led to an overestimation of protein content as described previously^23^. Protein content values reported for extracts where the amino acid nitrogen determination methods was used (Buckwheat: salt & sonic 4.7%, *C. crispus*: salt & sonic: 6.0%, Spelt: salt & sonic: 8.9%) report lower protein % values for generated extracts compared to those observed for protein values calculated using the Dumas method. The Dumas method of protein determination is based on sample nitrogen content^24^. The Dumas method determines protein indirectly by combusting a sample to convert the sample into gases, primarily CO_2_, NO_2_, N_2_, and H_2_O. These gases are passed through a column converting nitrous oxides to N_2_, which is then measured to determine the percentage nitrogen within the sample^25^. Using a known protein to nitrogen ratio the percentage nitrogen composition is converted to percentage protein composition. This is not the case for amino acid determination as the nitrogen content used to determine the protein content is based on the detected amino acid concentration^26^. Amino acid nitrogen uses the direct measurement of amino acid concentrations detected within each protein extract using the known percentage nitrogen composition of each individual amino acid and calculating the nitrogen composition for the sample as a whole. With this in mind, it is more likely that the amino nitrogen protein content is the most accurate representation of the protein content of the protein extracts. In cases where there is the potential for non-protein, nitrogen contamination, determination of protein content through direct measurement of amino acid nitrogen should be given priority over indirect measurements of protein content such as the Dumas method.

A major consideration when evaluating protein extracts is the quality of the protein extracted and how this improves compared to protein content & quality in crude biomass. A major determining factor for protein quality is the essential amino acid content (EAA). From a nutritional standpoint, the primary importance of a protein source is its ability to provide amino acids to the body^27^. Humans and animals in general cannot make EAAs and need to consume them in the diet. Thus, amino acid composition is a key consideration for determining protein quality, as proteins where EAAs are not present and balanced will not satisfy nutritional requirements.

The protein extracts generated in this work resulted in a consistent increase in EAAs compared to the crude biomass. The best results in terms of EAA concentration were obtained with enzymatic extraction of protein. This method increased the EAA content from 30.79 mg/g for buckwheat whole to 38.01 mg/g for the protein extract. The EAA content obtained when enzymatic hydrolysis was applied to *C. crispus* resulted in an increase in EAA content from 33.71 mg/g for whole *C. crispus* to 37.34 mg/g for the protein extract and the EAA content increased from 28.90 mg/g to 38.10 mg/g for the Spelt protein extract. Iso-electric precipitation applied to Buckwheat resulted in the greatest overall increase in EAA content in protein extracts (30.79 mg/g for whole Buckwheat to 77.75 mg/g for protein extract). However, the TAA content also increased, and the percentage of EAAs compared to TAA did not vary greatly when compared to the source biomass. This is important as proteins containing higher percentages of EAAs have better nutritional quality. However, EAA requirements vary between age groups, and health status. Fewer grams of total protein will be required to satisfy the dietary requirements where this is the case. This can be seen in the projected theoretical maximum dietary indispensable amino acid score (DIAAS) scores shown in Fig. 3. The theoretical maximum DIAAS takes the amino acid content of each source biomass and compares it to the FAO dietary requirements for 6 month - 3 year old children^28^ and determines how well a protein source would satisfy nutritional requirements assuming all available amino acids were absorbed. When compared to the theoretical maximum DIAAS, the protein extracts generated using enzymes, due to increased EAAs, have an increased projected theoretical maximum DIAAS value. This is best illustrated by the changes observed for Spelt. The Spelt protein extract generated using enzymes has a projected limiting amino acid lysine increase from 54% of dietary requirements to 92% of dietary requirements. However, enzymatic breakdown of proteins, is not specific to increase EAAs and the overall increase in EAAs may not be equally distributed, as shown by the variance in the differences between Buckwheat crude protein and Buckwheat protein enzymatic extract, in which some amino acids saw minor decreases in their relative satisfaction of dietary requirements (Leucine). Other amino acids saw major increases, like threonine, which increased from 144% to 225% of dietary requirements. For *C. crispus*, a decrease in the limiting amino acid histidine from 92% of dietary requirements in whole biomass to 72% of dietary requirements in the protein extract. In addition, the methods used to quantify amino acids are destructive to Cysteine, Methionine and Tryptophan. Therefore, there is a limitation to the value of the theoretical maximum DIAAS using this method.

Based on the theoretical maximum DIAAS, extracts generated using the enzymatic method show greater potential to act as nutritionally sufficient protein sources. For example, the *C. crispus* enzymatic protein extract has the potential to act as an excellent nutritional protein as its projected limiting amino acid, Histidine, is only slightly below the 75% threshold required to make a “source of good protein” claim^28^. For Spelt, lysine, the limiting amino acid, exceeds the 75% threshold and improves on lysine in the biomass as a source of “good protein”. In the case of the Buckwheat extract generated with enzymatic treatment, all of the detected amino acids have the potential to exceed to 100% required to make a claim of “excellent source of protein”.

In addition to how well a protein source may satisfy the nutritional requirements by providing EAAs, it is also important to consider how any compounds present within protein extracts can affect protein digestion^7^. Protein extracts generated in this work were assessed for their anti-nutritional factors including phenols, trypsin inhibitors and carbohydrates. There was no clear pattern in polyphenol content of protein extracts generated using the three different extraction methods from the three different biomasses. However, there was an increase in polyphenol content in protein extracts compared with the original biomasses. This is likely due to the high-water solubility of polyphenols. It is difficult to predict how this change in phenolic content would affect the quality of a protein extract, as phenolics reduce protein digestibility^29^ but are also beneficial beyond basic, human nutrition, due to antioxidant activities, which could increase the value of the protein depending on application^30^. It is also important to highlight the potential that some changes in detected phenol content may be due to the fact that Folin reagent also reacts with aromatic amino acids which may also be increased in the extraction process. A reduction in carbohydrate content was observed in protein extracts generated using enzymes compared to whole biomass. This difference is likely due to the carbohydrate hydrolysing enzymes reducing polysaccharides to oligosaccharides^31^. It is also worth noting that it is likely that the undetermined fraction of the total compositional analysis is carbohydrate content as this would be in line with the United States food and drug administration method for carbohydrate content determination. In terms of trypsin inhibition, only the Buckwheat Salt & Sonic extract resulted in decreases in trypsin activity which would result in decreased digestibility^32^. The observed reduction in trypsin activity would be sufficient to reduce the overall protein digestibility as less conversion of protein to di and tri peptides would occur over the same digestive period due to the reduction of enzyme activity. This is in line with the literature, in which Buckwheat has a strong association with trypsin activity inhibition^33^. Trypsin inhibition was not observed for any of the enzymatically generated extracts or the iso-electric precipitated extracts. This may be due to the fact that both methods incorporate a heating step and heat treatment is known to reduce anti-trypsin activity^16^.

The enzymatic protein extraction method employing the enzymes Viscozyme and Alcalase in sequence applied to Buckwheat and *C. crispus* biomass results in proteins of quality where significant quantities are generated. The quality and quantity of proteins obtained using this method is greater than when the salt & sonic and iso-electric precipitation methods are used on to the same biomass. In terms of extract yield, the enzymatic method results in protein extracts with the greatest yield of protein and the best quality proteins in terms of ratio of EAA: TAA. Furthermore, enzymatically generated protein extracts do not have a significant increase (P < 0.05) in anti-nutritional factors and have no significant impact (P < 0.05) on trypsin activity. Protein extracts made using enzymatic hydrolysis may be suitable for further testing and product development as alternative protein sources for nutritional and health benefits. However, the study is limited in that all results generated relate to in vitro studies. It is well know that in vitro methods for protein determination including amino acid analysis and the DUMAS method can underestimate and overestimate protein content, respectively^22^^,34^. Results generated in this study should be verified using ileal protein digestibility studies in pigs or rats.

## Methods

### Reagents and Biomass used in this study

1 M and 0.1 M sodium hydroxide (NaOH), (Fisher Scientific, Dublin, Ireland), 1 M and 0.1 M hydrochloric acid (HCl), (Fisher Scientific, Dublin, Ireland), Ammonium Sulphate ((NH_4_)_2_SO_4_), QuantiPro™ BCA Assay Kit (Merck, Dublin, Ireland), Viscozyme® L (Merck, Dublin, Ireland), Alcalase® Enzyme (VWR International, Dublin, Ireland). Molecular weight cut off (MWCO) tubing (3.5 kDa cut off), dialysis tubing, (Thermo Scientific, Dublin, Ireland), p-toluene-sulfonyl-L-arginine methyl ester (TAME),(Merck, Dublin, Ireland), Phenol, (Merck, Dublin, Ireland), Pancreatin, (Merck, Dublin, Ireland), Folin-Ciocalteu reagent, (Thermo Scientific, Dublin, Ireland), Tris/HCl buffer, (Merck, Dublin, Ireland),CaCl_2_, (Merck, Dublin, Ireland), soybean trypsin inhibitor, (Thermo Scientific, Dublin, Ireland).

Buckwheat was supplied by Redmond Fine Foods, Naas, Ireland. Organic Buckwheat Groats, whole and peeled were supplied sealed in plastic bags and contained 1 kg of buckwheat. The labelled composition of supplied buckwheat was Protein: 9.8 g/100 g, Fat: 1.7 g/100 g, Salt: <0.01 g/100 g, Carbohydrate: 71 g/100 g. SeaLac Limited, Kiltimagh, Mayo, Ireland supplied *Chondrus crispus* (Irish moss seaweed) in 12 kg bags. Seaweed was harvested from the west coast of Ireland, dried at low temperatures and milled into flakes ( < 100 mm). Dunany Flour, Drogheda, Ireland, supplied Spelt. 500 g whole, organic spelt berries were supplied to Teagasc in plastic bags. The label composition of supplied Spelt was 9.1 g/100 g, Fat: 2.1 g/100 g, Salt: 0.10 g/100 g, Carbohydrate: 61.5 g/100 g.

### Salt & Sonic Extraction Method

Proteins were extracted from Buckwheat*, C. crispus* and Spelt, independently, using the method previously^4^ with the following modifications. Briefly, crude biomass was diluted with Milli-Q water at a ratio of 1: 50 (w: v), (20 g in 1 L). This mixture was placed in a beaker in an elma-ultrasonic TI-H-10 ultrasonic bath (Elma Schmidbauer GmbH, Singen, Germany) set to a frequency of 35 kHz, and left to incubate for 1 hr. The mixture was subsequently removed from the ultrasonic bath and incubated overnight at 4 °C. Solid biomass was separated from the liquid supernatant by centrifugation at 10,000 x g in a Sorvall lynx 6000 centrifuge (Thermo Scientific, Osterode, Germany) for 1 hr at 4 °C. Milli-Q water (200 ml) was added to the solid fraction and ultra-sonication was repeated. Supernatants were pooled and ammonium sulphate was added to achieve an 80% saturation. The resulting solution was left to stir at 4 °C for 1 hr to allow for protein precipitation. The solution was centrifuged at 10,000 x g for 1 hr at 4 °C to collect precipitated proteins. Post centrifugation, the supernatant was discarded and collected precipitate was re-suspended into 250 ml of Milli-Q water. This precipitate and water mix was then passed through a 3.5 kDa molecular weight cut off (MWCO) dialysis tube (Fischer Scientific, USA) which was then placed into 3 litres of Milli-Q water and left to incubate overnight at 4 °C with stirring. Subsequently, the contents within the dialysis tubing were pooled and dried using a FD80GP freeze-drier (Cuddon Freeze Dry, New Zealand).

### Enzyme Extraction Method

Proteins were extracted from Buckwheat, *C. crispus* and Spelt using a combination of methods previously described^5,6^ with the pH and temperature of the enzymes modified, based on the manufacturers’ recommendations.

Crude biomass was diluted with Milli-Q water at a ratio of 1: 50 (w: v), (20 g of biomass to 1 L of Milli-Q water). The pH of the mixture was adjusted to pH 5 using 1 M HCL. The enzyme Viscozyme L (≥100 FBGU/g) was added to this mixture at 3% (w/v) and the solution was placed in a Cole-Parmer® SI-200 Series Stuart Shaking Incubator (Cole-Parmer Instrument Co., St. Neots, United Kingdom) set to 45 °C, 150 rpm. The mixture was incubated for 3 hr with the pH rising to pH 6 over the course of the incubation. The pH of the mixture was then adjusted to pH 8.5 using 1 M NaOH and Alcalase (≥5 U/g) was added to the mixture at 3% (w/v). The mixture was incubated at 60 °C for 3 h with shaking at 150 rpm with the pH falling to pH 7.5 over the course of the incubation. Once incubation was completed, the biomass and enzyme mixture was placed into a water bath set to 90 °C and incubated for 10 minutes to deactivate the active enzymes. Following this, the biomass mixture was centrifuged at 8,000-x g for 10 minutes at 4 °C to separate the remaining biomass from the supernatant. The supernatant was collected, and freeze-dried, in a FD80GP freeze-drier (Cuddon Freeze Dry, New Zealand), to form the final protein extract.

### Iso-Electric Extraction Method

Proteins were extracted from Buckwheat, *C. crispus* and Spelt using the methods previously described^4,7^.

Crude biomass was diluted with Milli-Q water at a ratio of 1:50 (w: v), (20 g of biomass to 1 L of Milli-Q water). The mixture was left to stir for 1 hr on a heated stir plate set to 30 °C. The pH was increased using 1 M NaOH to the maximum pH in order to get maximum solubility of the specific biomass. Once the desired maximum pH of pH 13 was achieved, the sample was left to incubate for 1 hr with stirring at 30 °C. The biomass mix was subsequently centrifuged at 8000 x g for 10 minutes to separate biomass from the protein-containing supernatant. The supernatant was collected, and the pH was reduced to the lowest pH value, pH 3, in order to get minimum protein solubility for each specific biomass using HCL. Subsequently, the solution was left to incubate for 30 minutes at room temperature with stirring to allow for protein precipitation. The solution was centrifuged at 8000 x g for 10 minutes at 4 °C to separate the precipitated protein from solution. Once the precipitate was collected, the supernatant was discarded, and the precipitate was re-suspended in 250 ml of Milli-Q water, frozen and freeze-dried using a FD80GP freeze-drier (Cuddon Freeze Dry, New Zealand).

### Compositional Analysis

Sample moisture content was determined using a TGM 800 Thermogravimetric Analyser (LECO Corp., MI, USA) and an adapted version of method AOAC 925.10^35^. Briefly, a tin foil dish containing the protein extract (grams) or whole biomass (grams) was placed in the TGM 800 Thermogravimetric Analyzer. A blank reference dish without sample was used as a control. Samples were left until the samples reached a constant weight and the temperature exceeded 100 °C.

Determination of sample nitrogen was achieved according to AOAC method 992.15. Briefly, nitrogen content was determined by processing 100 mg of sample through the LECO FP628 Nitrogen Analyser (LECO Corp., MI, USA) which combusts samples at 850 °C in the presence of pure oxygen to determine sample nitrogen content. This nitrogen content was then used to calculate protein content using the following nitrogen to protein conversion factors: Spelt, 5.54^36^, *C. crispus*, 3.55^12^, Buckwheat, 5.94^36^. This protein determination was used to calculate the protein extraction yield using the following equation:

$${Extraction\; Yield}\left( \% \right)=\frac{{Extract\; Total\; Dry\; matter\; protein}(G)}{{Crude\; Biomass\; Total\; Dry\; Matter\; Protein}(G)}\times 100$$

Sample fat content was determined according to AOAC Method 2008.06^37^ using the Oracle Rapid NMR Fat Analyzer (CEM Corp., Charlotte, NC, USA). Briefly, this was achieved by taking the samples directly from the TGM 800 Thermogravimetric Analyser post moisture content determination^35^. Samples of crude biomass averaging 0.5 g and extracts averaging 0.2 g were placed between two glass fibre pads and heated at 40 °C on a heating block for 40 min. Samples were then placed into Oracle Rapid NMR Fat Analyzer individually and their fat content was determined using microwave NMR to determine fat content as a percentage of the total sample.

Ash content was determined using AOAC method 942.05^38^ with modifications (samples were left in the furnace overnight). Briefly, an average of 1 g of whole biomass and 0.5 grams of protein extracts were placed, independently, into pre-weighed and marked ceramic crucibles that were subsequently weighed and placed on a hot plate for 3 h at 100 °C until samples were charred. Samples were then transferred to a muffle furnace and heated overnight at 540 °C to reduce samples to ash. Samples and crucibles were retrieved and placed into a desiccator and left to cool. Once samples were cool and at room temperature, the crucibles were re-weighed, and the percentage ash content was determined by calculating the difference in sample weight prior and post incineration, less the weight of the ceramic crucible.

Sample fibre was quantified using the AOAC 985.29 method performed using the ANKOM TDF Fibre Analyzer (ANKOM Technology, NY, USA*)*. Briefly, samples were placed in filter-bottomed bags, which were then inserted into the ANKOM TDF Fibre Analyzer and samples were exposed to a series of enzyme hydrolyses and pH condition variations to remove non-fibre from the samples. Post treatment, samples were dried and tested for remaining protein and ash content, which was subtracted from the final sample dry weight in order to determine the fibre content. This was repeated in triplicate for all raw biomass samples.

Total Amino acid content was determined using an ultra-high-performance liquid chromatographic–diode array (UHPLC-DAD) method^39^. Briefly, 6 M HCl was added to the dried samples (0.210 g) and used for hydrolysis at 100 °C for 24 h. Samples were dried in a vacuum centrifuge (SpeedVac RVC 2-33 CDplus (Martin Christ Gefriertrocknungsanlagen GmbH, Osterod, Germany). Samples were reconstituted in 0.2 ml water and loaded into vials. Results were obtained using UHPLC-DAD using an Agilent 1290 UHPLC system after derivatization of amino groups. Two different system suitability tests (SSTs) were measured along with the samples to verify the calibration. Analysis was performed in duplicate. Xell AG (Göttingen, Germany) carried out the amino acid analysis.

Amino acid nitrogen (AAN) determination was based on the method described by Fujihara and colleagues^36^. In this method sample nitrogen content is determined via calculation based on the detection of amino acids as described earlier with nitrogen content being derived from the nitrogen found in each amino acid. The sample nitrogen content was then converted to protein using the nitrogen to protein conversion factor conversion 6.25 as protein to nitrogen factors found in the literature are based on the potential for non-protein nitrogen which is not present in direct amino acid analysis. This analysis was performed in duplicate.

### Theoretical Maximum Dietary Indispensable Amino Acid Score (DIAAS)

The theoretical maximum DIAAS was calculated based on amino acid quantification of each protein extract. Following the quantification the degree to which the amino acids could satisfy dietary requirements as provided by the FAO using the following equation:

$${DIAAS}=\frac{{mg\; of\; essential\; amino\; acid\; per\; gram\; of\; extract\; protein}}{{mg\; of\; essential\; amino\; acid\; per\; gram\; of\; refference\; protein}}$$

### Determination of anti-nutritional factors

Total phenolic content of protein extracts was determined using the Folin-Ciocalteu assay^15^. Briefly, samples were tested by loading 20 µL of the dissolved sample into 1.58 ml of Milli-Q water in a 2 ml test-tube to which 100 µL of Folin-Ciocalteu reagent was added. Samples were mixed and incubated for 5 min. Sodium carbonate solution (300 µL of a 1.9 M solution) was subsequently added to each tube and tubes mixed and incubated at 40 °C for 30 min. Once incubation was complete, 1 ml of each test sample was loaded into a cuvette and the absorbance was measured at 765 nm using a photospectrometer. Absorbance values from samples were then compared to the absorbance values of a blank and a standard curve of Gallic acid (GA) comprising the following concentrations 0 mg/ml, 50 mg/ml, 100 mg/ml, 150 mg/ml 250 mg/ml, 500 mg/ml. Following this, the value of Gallic acid equivalents (GAEs) for each sample was determined and corrected based on sample dilutions to determine the total phenolic content expressed as GAE/mg sample. Analysis was carried out in triplicate for each source biomass and protein extract.

Carbohydrate content was determined using the Dubois method based on the methods described previously^16,40^. Samples were assessed by incubating samples in 12 M H_2_SO_4_ at 37 °C for 1 hour. Following this, 50 µL of sample and glucose standard was loaded into a 96 well plate. To each test and control sample, 150 µL of concentrated (98%) H_2_SO_4_ was added to each well and the mixture was incubated with shaking for 30 min. Subsequently, 30 µL of 5% phenol was added to each test or control well and the 96 well plate was heated for 5 min at 90 °C. After cooling, samples absorbance was measured at 490 nm using a BMG LABTECH FLUOstar Omega plate reader. Sample absorbance values were compared to the glucose standard to determine the carbohydrate content of each sample.

Trypsin inhibitor activity of the protein extracts was determined by measuring the conversion rate of p-toluene-sulfonyl-L-arginine methyl ester (TAME) at 25 °C and pH 8.2 in the presence of the extracts. Following this, pancreatin (8U/mg (Merck, Dublin, Ireland)) was mixed with the protein extracts to determine if the rate of TAME conversion was significantly reduced. This is indicative of a reduction in trypsin activity. Results obtained were compared to the known trypsin inhibitor - soybean trypsin inhibitor (Thermo Scientific, Dublin, Ireland)). Based on the method previously described^41,42^, samples were tested by dissolving extract samples at a concentration of 2.5 mg/ml in Milli-Q water. 50 µL of dissolved sample was mixed with 2.6 mL of assay buffer containing 46 mM Tris/HCl buffer, 11.5 mM CaCl_2_ at pH 8.1, 0.3 mL of 10 mM TAME solution, and 50 µL of 0.5 mg/ml pancreatin was placed in a quartz cuvette. The cuvette containing the sample, pancreatin, and TAME mix was incubated in the Molecular Devices SpectraMax ABS plus (Molecular Devices, San Jose, USA) at 25 °C for 10 minutes. Absorbance at 247 nm was measured at 30-second intervals. Absorbance was also determined for uninhibited pancreatin and pancreatin in the presence of a soybean-based trypsin inhibitor at 2.5 mg/ml and a control blank. These absorbance values were used to determine trypsin activity by measuring the change in absorbance over a 5 min period, which is used to determine trypsin activity as the rate of TAME digestion at 25 °C.

### Statistical analysis

All tests were performed in duplicate on three replicate samples (*n* = 6), with the exception of fibre analysis, which was only performed on crude biomass, and amino acid testing of iso-electric extracts, which were performed in duplicate on a single sample (*n* = 2). Analysis regarding extracts generated using the salt & sonic method were performed in duplicate on two replicate samples (*n* = 4). All statistical analysis were performed using GraphPad Prism 9 using a two-way ANOVA with a Tukey multiple comparison test, with statistical significance being determined as a *P*-value less than 0.05. For protein extraction yield, data was analysed within a protein extraction method, significant difference to sonic & salt extraction data is depicted by letter A, significant difference to enzymatic extraction is depicted by letter B, significant difference to iso-electric extraction is depicted by letter C. For measurement of protein content within a protein extraction method, matching letters indicate no statistically significant difference. For amino acid content, data was analysed compared to crude biomass with * indicating significant differences. For phenolic content, data was analysed for each protein extraction method. Significant difference to crude biomass data is depicted by the letter A. Significant difference to salt & sonic extract data is depicted by letter B. Significant difference to enzymatic extraction method is depicted by letter C. Significant difference to iso-electric extraction method is depicted by letter D. For carbohydrate content, data were analysed between each protein extraction method. Significant difference to crude biomass data are depicted by letter A, significant difference to salt & sonic extract data are depicted by letter B, significant difference to enzymatic extraction method is depicted by letter C, significant difference to iso-electric extraction method is depicted by letter D. For trypsin inhibitor activity, data was analysed to determine statistical significances and is denoted by *, between extracts when compared to trypsin levels in uninhibited pancreatin.

## Supplementary information

Supplemental Material

## Data Availability

Data generated is available from the corresponding author.
