# Supplementary material for: Protein extraction from Buckwheat, Chondrus crispus, and Spelt and assessment of nutritional benefits and limitations in vitro
Source: NPJ Sci Food. 2025 Sep 24;9:193. doi: 10.1038/s41538-025-00540-6 (PMC12460845; doi:10.1038/s41538-025-00540-6)
Supplement: Supplementary file 1 — Supplemental Material [file 41538_2025_540_MOESM1_ESM.pdf]

Supplemental Material:

Full Compositional Data:

Buckwheat:

Crude: Total mass: 20g Protein: 8.32%, Moisture: 13.11%, Fat: 2.60%, Ash: 1.85%, Carbohydrate: 15.08%, Phenols: 0.34%, Fibre: 3.10%, Undetermined (Assumed Carbohydrates): 55.60%, FDA Carbohydrate Calculation: 73.78%

Salt & Sonic: Total mass: 2.6g Protein: 4.23%, Moisture: 9.07%, Fat: 0.79%, Ash: 0.16%, Carbohydrate: 8.73%, Phenols: 0.71%, Undetermined (Assumed Carbohydrates): 76.31%, FDA Carbohydrate Calculation: 85.04%

Enzyme Extract: Total mass: 4.7g Protein: 9.51%, Moisture: 5.31%, Fat: 0.40%, Ash: 5.39%, Carbohydrate: 16.50%, Phenols: 0.32%, Undetermined (Assumed Carbohydrates): 62.56%, FDA Carbohydrate Calculation: 79.06%

Iso-electric Precipitation: Total mass: 1.2g Protein: 23.71%, Moisture: 10.16%, Fat: 3.58%, Ash: 11.15%, Carbohydrate: 26.77%, Phenols: 0.16%, Undetermined (Assumed Carbohydrates): 24.47%, FDA Carbohydrate Calculation: 51.24%

***C.crispus*:**

Crude: Total mass: 20g Protein: 8.62%, Moisture: 12.47%, Fat: 0.35%, Ash: 23.77%, Carbohydrate: 27.19%, Phenols: 0.54%, Fibre: 43.80, Undetermined (Assumed Carbohydrates):16.75%, FDA Carbohydrate Calculation: 54.25%

Salt & Sonic: Total mass: 1.4g, Protein: 5.77%, Moisture: 4.58%, Fat: 0.40%, Ash: 2.09%, Carbohydrate: 32.87%, Phenols: 1.01%, Undetermined (Assumed Carbohydrates): 53.28%, FDA Carbohydrate Calculation: 86.15%

Enzyme Extract: Total mass: 8.9g, Protein: 10.10%, Moisture: 9.32%, Fat: 0.99%, Ash: 23.38%, Carbohydrate: 18.94%, Phenols: 0.96%, Undetermined (Assumed Carbohydrates): 36.31%, FDA Carbohydrate Calculation: 55.25%

Iso-electric Precipitation: Total mass: 0.7g, Protein: 11.61%, Moisture: 15.97%, Fat: 1.49%, Ash: 33.08%, Carbohydrate: 20.77%, Phenols: 1.04%, Undetermined (Assumed Carbohydrates): 16.04%, FDA Carbohydrate Calculation: 36.81%

Spelt:

Crude: Total mass: 20g, Protein: 8.32%, Moisture: 10.92%, Fat: 2.05%, Ash: 1.68%, Carbohydrate: 23.99%, Phenols: 0.02%, Fibre: 11.00%, Undetermined (Assumed Carbohydrates): 42.02%, FDA Carbohydrate Calculation: 77.01%

Salt & Sonic: Total mass: 4.2g, Protein: 8.26%, Moisture: 7.65%, Fat: 1.33%, Ash: 0.30%, Carbohydrate: 33.60%, Phenols: 0.44%, Undetermined (Assumed Carbohydrates): 48.42%, FDA Carbohydrate Calculation: 82.02%

Enzyme Extract: Total mass: 3.3g, Protein: 11.03%, Moisture: 9.54%, Fat: 1.73%, Ash: 10.27%, Carbohydrate: 23.87%, Phenols: 0.66%, Undetermined (Assumed Carbohydrates): 42.90%, FDA Carbohydrate Calculation: 66.77%

Iso-electric Precipitation: Total mass: 0.8g, Protein: 9.74%, Moisture: 10.38%, Fat: 6.05%, Ash: 2.40%, Carbohydrate: 30.13%, Phenols: 0.12%, Undetermined (Assumed Carbohydrates): 41.18%, FDA Carbohydrate Calculation: 71.31%

Supplementary Table 1: Amino Acid Composition of Spelt Protein in Crude Biomass and Protein extracts.

Source	Crude Spelt Protein (mg/g)	Spelt Salt & Sonic Protein Extract (mg/g)	Spelt Enzyme Protein Extract (mg/g)	Spelt Iso-Electric Protein Extract (mg/g)
**Cys**	N/A	N/A	N/A	N/A
**Cys-Cys**	N/A	0.76	N/A	N/A
**His**	2.24	1.83	2.62	2.64
**Ile**	3.27	2.91	4.58	3.57
**Leu**	6.67	6.28	8.03	7.05
**Lys**	2.78	2.35	4.51	4.53
**Met**	N/A	0.72	N/A	N/A
**Phe**	4.27	4.27	5.15	4.32
**Thr**	3.00	2.53	4.90	3.47
**Trp**	N/A	N/A	N/A	N/A
**Tyr**	1.69	2.13	1.99	2.29
**Val**	4.22	3.84	6.31	5.12
Ala	3.65	3.39	6.12	4.93
Arg	4.15	3.64	5.54	6.25
Asn	N/A	N/A	N/A	N/A
Asp	5.25	4.30	9.39	7.27
Gln	N/A	N/A	N/A	N/A
Glu	30.79	28.11	21.36	19.69
Gly	3.80	3.26	5.81	5.08
Pro	9.13	9.98	9.29	6.25

Ser	4.23	3.36	5.86	4.10
Cit	N/A	N/A	N/A	N/A
Eta	N/A	N/A	N/A	N/A
HyPro	N/A	N/A	0.21	N/A
Orn	N/A	N/A	N/A	N/A
Tau	N/A	N/A	N/A	N/A
∑EAA	28.90	27.62	38.10	32.98
∑TAA	90.03	83.87	101.67	86.55
% EAA	32%	33%	37%	38%

Supplementary table 1 Legend: Amino Acid Composition of spelt Protein in Crude Biomass and Protein Extracts. Bold text indicates essential amino acids, ∑EAA = sum of essential amino acids, ∑TAA = sum of total amino acids, EAA% = essential amino acids as percentage of total amino acids, N/A: not detected or not detected in all replicates. Data represents an average of duplicate tests.

Supplementary Table 2: Amino Acid composition of Chondrus crispus protein in Crude Biomass and Protein Extracts.

Source	Crude Chondrus crispus Protein (mg/g)	Chondrus crispus Salt & Sonic Protein Extract (mg/g)	Chondrus crispus Enzyme Protein Extract (mg/g)	Chondrus crispus Iso-Electric Protein Extract (mg/g)
**Cys**	N/A	N/A	N/A	N/A
**Cys-Cys**	N/A	N/A	N/A	N/A
**His**	1.51	1.51	1.51	1.51
**Ile**	3.59	3.59	3.59	3.59
**Leu**	6.06	6.06	6.06	6.06
**Lys**	5.63	5.63	5.63	5.63
**Met**	N/A	N/A	N/A	N/A
**Phe**	4.08	4.08	4.08	4.08
**Thr**	4.77	4.770	4.77	4.77
**Trp**	N/A	N/A	N/A	N/A
**Tyr**	2.54	2.54	2.54	2.54
**Val**	4.79	4.79	4.79	4.79
Ala	6.02	6.02	6.02	6.02
Arg	6.56	6.56	6.56	6.56
Asn	N/A	N/A	N/A	N/A
Asp	10.28	10.28	10.28	10.28
Gln	N/A	N/A	N/A	N/A
Glu	8.08	8.08	8.08	8.08
Gly	6.09	6.09	6.09	6.09
Pro	4.21	4.21	4.21	4.21

Ser	4.56	4.56	4.56	4.56
Cit	N/A	N/A	N/A	N/A
Eta	N/A	N/A	N/A	N/A
HyPro	N/A	N/A	N/A	N/A
Orn	0.49	0.49	0.49	0.49
Tau	1.96	1.96	1.96	1.96
∑EAA	33.71	22.97	37.34	35.71
∑AA	82.19	54.40	90.81	93.54
% EAA	41.1%	42.2%	41.1%	38.2%

Supplementary Table 2 Legend: Amino Acid Composition of Chondrus crispus Protein in Crude Biomass and Protein Extracts. Bold text indicates essential amino acids, ∑EAA = sum of essential amino acids, ∑TAA = sum of total amino acids, EAA% = essential amino acids as percentage of total amino acids, N/A: not detected or not detected in all replicates. Data represents an average of duplicate tests.

Supplementary Table 3: Amino Acid composition of Buckwheat Protein in Crude Biomass and Protein Extracts.

Source	Crude Buckwheat Protein (mg/g)	Buckwheat Salt & Sonic Protein Extract (mg/g)	Buckwheat Enzyme Protein Extract (mg/g)	Buckwheat Iso-Electric Protein Extract (mg/g)
**Cys**	N/A	N/A	N/A	N/A
**Cys-Cys**	N/A	N/A	N/A	N/A
**His**	2.08	0.91	2.23	5.61
**Ile**	3.20	1.47	4.29	8.82
**Leu**	5.88	2.82	6.39	14.90
**Lys**	5.26	2.43	5.47	12.24
**Met**	N/A	N/A	N/A	N/A
**Phe**	3.98	1.90	4.28	11.13
**Thr**	3.51	1.61	6.03	8.18
**Trp**	N/A	N/A	N/A	N/A
**Tyr**	1.68	1.08	3.04	5.67
**Val**	4.27	1.95	6.27	11.21
Ala	4.10	1.98	6.45	9.26
Arg	7.82	3.40	5.68	21.25
Asn	N/A	N/A	N/A	N/A
Asp	8.85	4.23	12.09	22.40
Gln	N/A	N/A	N/A	N/A
Glu	14.83	6.53	6.86	37.26
Gly	5.11	2.69	6.73	13.36
Pro	3.16	1.66	3.67	8.61
Ser	4.18	1.94	6.61	10.50
Cit	N/A	N/A	N/A	N/A
Eta	N/A	N/A	N/A	N/A

HyPro	N/A	N/A	0.29	0.51
Orn	N/A	N/A	N/A	N/A
Tau	N/A	N/A	N/A	N/A
∑EAA	30.79	14.16	38.01	77.75
∑AA	78.97	36.60	86.39	200.90
% EAA	39.1%	38.7%	44.0%	38.7%

Supplementary Table 3 Legend: Amino Acid Composition of Buckwheat Protein in Crude Biomass and Protein Extracts. Bold text indicates essential amino acids, ∑EAA = sum of essential amino acids, ∑TAA = sum of total amino acids, EAA% = essential amino acids as percentage of total amino acids, N/A: not detected or not detected in all replicates. Data represents an average of duplicate tests.

Supplementary Table 4: Theoretical Max DIAAS Values for Crude Biomass and Salt & Sonic Extract.

Amino Acids	His	Ile	Leu	Lys	Thr	Trp*	Val	SAA (Cys + Met)*	AAA (Phe + Tyr)
Dietary Requirement Scoring Pattern (mg/1g of protein)	20.00	32.00	66.00	57.00	31.00	8.50	43.00	27.00	52.00
Crude Spelt Protein (% Dietary requirement)	124.65	113.53	112.18	54.13	107.55	-	108.86	-	127.32
Spelt Salt & Sonic Protein Extract (% Dietary requirement)	109.32	108.53	113.41	49.24	97.20	-	106.63	65.52	146.59
Spelt Enzyme Protein Extract (% Dietary requirement)	128.57	140.70	119.28	91.75	152.12	-	142.47	-	136.21
Spelt Iso-Electric Protein Extract (% Dietary requirement)	152.35	128.94	123.33	91.80	129.20	-	137.64	-	146.86
Crude Chondrus crispus Protein (% Dietary requirement)	91.69	136.14	111.82	120.03	187.37	-	135.26	-	154.61
Chondrus crispus Salt & Sonic Protein Extract (% Dietary requirement)	88.80	148.78	127.35	106.31	186.15	-	140.92	-	180.67
Chondrus crispus Enzyme Protein Extract (% Dietary requirement)	72.21	159.58	116.62	86.20	193.20	-	155.29	53.64	150.80
Chondrus crispus Iso-Electric Protein Extract (% Dietary requirement)	84.92	139.61	105.73	109.18	179.56	-	131.32	-	146.11
Crude Buckwheat Protein (% Dietary requirement)	132.09	128.18	113.34	117.06	143.76	-	127.13	-	138.37

Buckwheat Salt & Sonic Protein Extract (% Dietary requirement)	124.08	125.33	116.86	116.37	142.09	-	123.90	-	156.23
Buckwheat Enzyme Protein Extract (% Dietary requirement)	129.25	155.02	112.02	111.17	225.22	-	168.77	-	163.10
Buckwheat Iso-Electric Protein Extract (% Dietary requirement)	139.57	137.13	112.35	106.84	131.40	-	129.78	-	160.85

Supplementary Table 4 Legend: Data is reported as percentage of the scoring pattern. Data represents an average of duplicate tests. Green Values indicate greater than 100%, Yellow Values indicate results less than 100%, Black values indicates that no value was reported or that not all replicates reported a value, and Red Underline indicates potential to be the limiting amino acid. *, Indicate amino acids that are likely to be degraded as a result of hydrolysis conditions.
